# Supplementary material for: Identification of Loci Associated with Enhanced Virulence in Spodoptera litura Nucleopolyhedrovirus Isolates Using Deep Sequencing
Source: Viruses. 2019 Sep 17;11(9):872. doi: 10.3390/v11090872 (PMC6783950; doi:10.3390/v11090872)
Supplement: Supplementary file 1 [file viruses-11-00872-s001.zip › viruses-564066 revised supplementary - to proofreading/supplementary Figure S1, S2, S3 and Table S1.pdf]

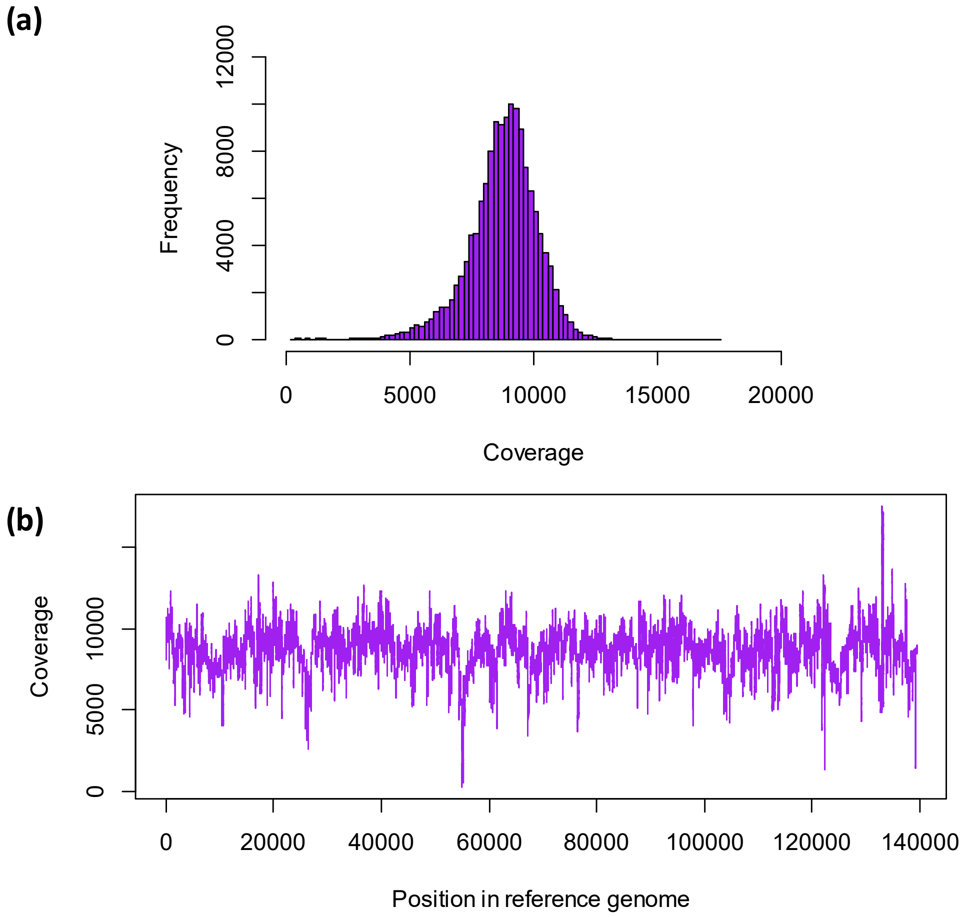

Figure S1. Coverage resulting from the mapping of SpltNPV-G2b to the SpltNPV-G2 reference sequence is shown. (**a**) A histogram of coverage per nucleotide is given. (**b**) The coverage per position in the SpltNPV-G2 reference is shown.

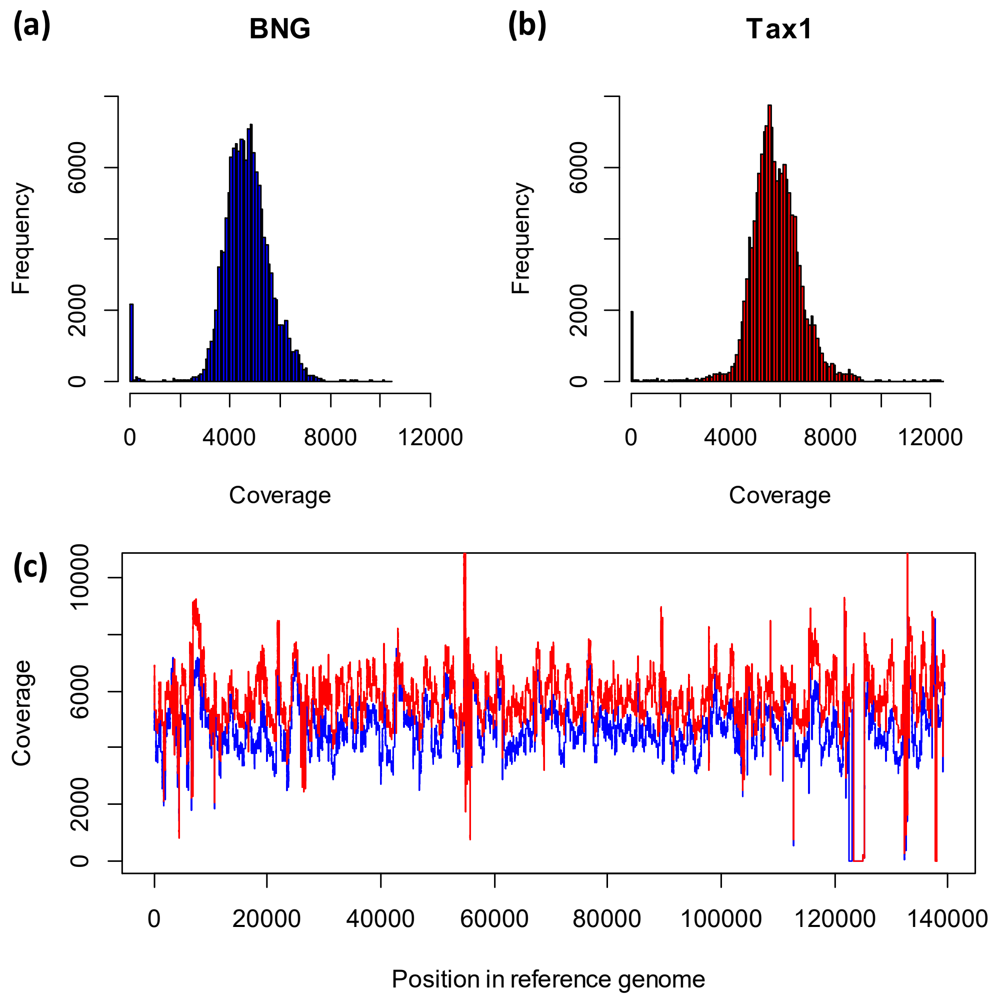

Figure S2. (**a**) The coverage per nucleotide position for the resequencing analysis is shown in histograms for isolate SpltNPV-Pak-BNG. (**b**) The coverage for SpltNPV-Pak-TAX1 is given. Note the similarity of the distributions for SpltNPV-Pak-BNG and SpltNPV-Pak-TAX1, and that approximately 2000 positions have little or no coverage. (**c**) The coverage per position in the SpltNPV-G2 reference is shown in blue for SpltNPV-Pak-BNG and in red for SpltNPV-Pak-TAX1. Note the two regions in which there is no coverage.

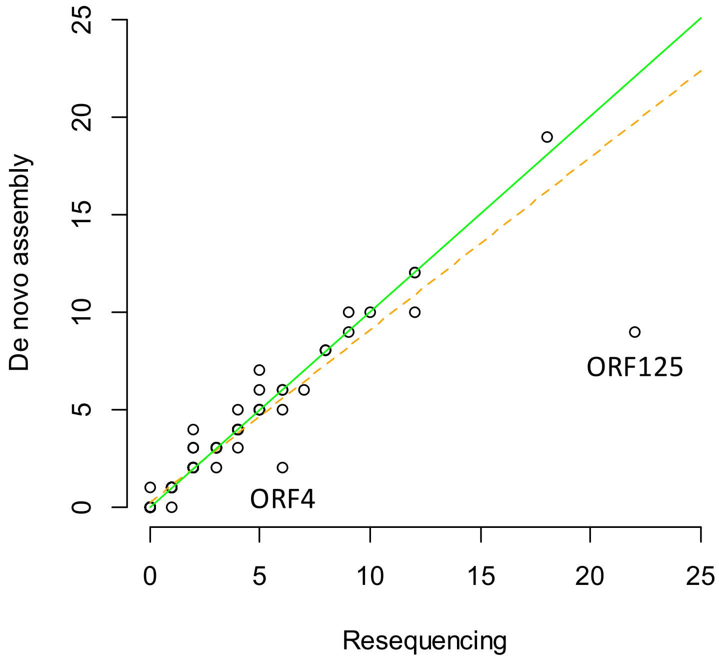

Figure S3. The total number of unique synonymous and nonsynonymous mutations per ORF (i.e., the sum of unique mutations in SpltNPV-Pak-BNG and SpltNPV-Pak-TAX1 when analyzed by resequencing, or all mutations identified in the de novo assembly approach) is plotted for the resequencing (x-axis) and de novo assembly (y-axis) approaches. The dotted orange line is the model 2 regression line using all data, whilst the green line is the model 2 regression line excluding ORF125 (data point labelled), which appears to be an outlier. For all data, the slope of the regression line is 0.833, whereas without ORF125 the slope is 1.004. With the exception of ORF4 and ORF125, there seems to be very good agreement between the two deep sequencing analysis methods.

Table S1. Predicted and observed restriction fragment length polymorphism band lengths, with predictions based on the de novo assembled sequences. All band lengths are in base pairs. ND indicates "not distinguished" for the empirical data, suggesting that a band is likely present but could not be positively identified because its length is similar to another band.

BNG S9 de novo		TAX1 S10 de novo	
**Predicted**	**Observed**	**Predicted**	**Observed**
16028	15000	16021	15000
11420	10500	11386	10500
10496	ND	10480	10000
10220	10000	10217	ND
9386	9000	9393	8250
7918	8000	7765	8000
7458	7500	7458	7500
6779	6500	6795	6500
5533	5250	5675	5500
5389	ND	5250	5250
5250	ND	5078	5000
5010	4900	5058	ND
4621	4750	4617	4500
4613	4500	4614	ND
4493	4250	4421	4250
2612	2500	2612	2500
2423	2300	2423	2300
2370	2250	2370	ND
2266	2100	2260	2250
2186	2000	2198	2100
1941	1750	1938	2000
1886	1600	1886	1750
1357	1300	1663	1600
1171	1200	1357	1300
1062	1100	1062	1200
1061	ND	1061	1100
976	No longer visible	976	No longer visible
351		391	
243		351	
222		243	
218		222	
196		218	
		196	
