# Supplementary material for: Identification of Loci Associated with Enhanced Virulence in Spodoptera litura Nucleopolyhedrovirus Isolates Using Deep Sequencing
Source: Viruses. 2019 Sep 17;11(9):872. doi: 10.3390/v11090872 (PMC6783950; doi:10.3390/v11090872)
Supplement: Supplementary file 1 [file viruses-11-00872-s001.zip › viruses-564066 revised supplementary - to proofreading/Supplementary Materials/Dataset_3/Dataset 3.pdf]

Dataset 3: Manual curation of within-isolate polymorphisms. Position is the nucleotide position in the corresponding de novo consensus sequence, length is size of the mutation, freq. is the frequency of the mutation (count/coverage), F./R. is the ratio of reads mapping in the forward to reverse orientations, avg. score is the average Phred score, and manual curation gives the results of inspections of the read mappings.

| Virus | Position | Class | Length | Reference | New | Count | Coverage | Freq. | F./R. | Avg. Score | Manual curation |
|---|---|---|---|---|---|---|---|---|---|---|---|
| BNG | 10396 | Deletion | 1 | T | - | 221 | 3915 | 5.645 | 0.457 | 36.742 | Artefact repeated T stretch |
| | 10396 | Insertion | 1 | - | T | 135 | 3867 | 3.491 | 0.431 | 37.733 | Artefact repeated T stretch |
| | 14399 | Insertion | 1 | - | T | 1277 | 3392 | 37.647 | 0.487 | 37.288 | Artefact repeated T stretch |
| | 14399 | Deletion | 1 | T | - | 84 | 3503 | 2.398 | 0.447 | 36.702 | Artefact repeated T stretch |
| | 29118 | Deletion | 1 | A | - | 106 | 3447 | 3.075 | 0.433 | 36.434 | Artefact repeated A stretch |
| | 35000 | SNV | 1 | C | A | 155 | 3881 | 3.994 | 0.442 | 37.465 | Authentic |
| | 35202 | SNV | 1 | C | T | 1623 | 3455 | 46.975 | 0.500 | 37.212 | Authentic |
| | 40062 | Deletion | 1 | A | - | 173 | 2940 | 5.884 | 0.383 | 36.457 | Artefact repeated A stretch |
| | 41180 | SNV | 1 | G | A | 60 | 2910 | 2.062 | 0.478 | 36.967 | Authentic |
| | 45843 | Deletion | 3 | TAG | - | 92 | 3669 | 2.507 | 0.472 | 36.935 | Artefact repeated T stretch |
| | 54562 | Insertion | 1 | - | A | 792 | 3571 | 22.179 | 0.492 | 37.556 | Artefact repeated A stretch |
| | 54562 | Deletion | 1 | A | - | 123 | 3818 | 3.222 | 0.485 | 36.333 | Artefact repeated A stretch |
| | 68688 | Deletion | 2 | AC | - | 168 | 3577 | 4.697 | 0.451 | 37.015 | Artefact repeat region |
| | 94442 | Deletion | 1 | T | - | 245 | 4343 | 5.641 | 0.436 | 36.878 | Artefact repeated T stretch |
| | 97803 | SNV | 1 | C | T | 217 | 3219 | 6.741 | 0.271 | 37.230 | Artefact repeat region |
| | 97957 | Deletion | 3 | TCG | - | 364 | 1382 | 26.339 | 0.403 | 37.357 | Artefact repeat region |
| | 97961 | Deletion | 14 | CCACGTTCGATCAT | - | 382 | 1354 | 28.213 | 0.413 | 37.370 | Artefact repeat region |
| | 97977 | SNV | 1 | A | C | 562 | 2375 | 23.663 | 0.379 | 37.527 | Artefact repeat region |
| | 97977 | Deletion | 2 | AT | - | 341 | 2427 | 14.050 | 0.482 | 37.388 | Artefact repeat region |
| | 97978 | Deletion | 1 | T | - | 1505 | 2427 | 62.011 | 0.387 | 36.747 | Artefact repeat region |
| | 97979 | SNV | 1 | A | T | 558 | 2467 | 22.619 | 0.376 | 37.427 | Artefact repeat region |
| | 104686 | Insertion | 1 | - | A | 133 | 4810 | 2.765 | 0.477 | 37.534 | Artefact repeated A stretch |
| | 110733 | Deletion | 1 | T | - | 313 | 4491 | 6.969 | 0.427 | 36.572 | Artefact repeated T stretch |
| | 110733 | Insertion | 1 | - | T | 98 | 4461 | 2.197 | 0.415 | 37.153 | Artefact repeated T stretch |
| | 111904 | SNV | 1 | C | T | 407 | 3822 | 10.649 | 0.493 | 37.314 | Authentic |
| | 112846 | Insertion | 1 | - | T | 230 | 3989 | 5.766 | 0.434 | 37.626 | Artefact repeated T stretch |
| | 112846 | Deletion | 1 | T | - | 105 | 4049 | 2.593 | 0.458 | 36.724 | Artefact repeat region |
| | 116029 | Deletion | 3 | GAT | - | 89 | 4360 | 2.041 | 0.439 | 36.647 | Artefact repeated GAT sequence |
| TAX1 | 3227 | Deletion | 2 | GT | - | 386 | 4894 | 7.887 | 0.471 | 35.933 | Artefact repeated region |
| | 10393 | Insertion | 1 | - | T | 110 | 4637 | 2.372 | 0.459 | 37.436 | Artefact repeated T stretch |
| | 16680 | Deletion | 2 | TA | - | 451 | 4992 | 9.034 | 0.453 | 36.230 | Artefact repeated region |
| | 16680 | Insertion | 2 | - | TA | 263 | 4463 | 5.893 | 0.389 | 37.736 | Artefact repeated region |
| | 16680 | Deletion | 4 | TATA | - | 109 | 5245 | 2.078 | 0.393 | 36.770 | Artefact repeated region |
| | 26850 | Deletion | 1 | T | - | 238 | 5251 | 4.532 | 0.476 | 36.882 | Artefact repeated T stretch |
| | 26850 | Insertion | 1 | - | T | 106 | 5185 | 2.044 | 0.482 | 37.585 | Artefact repeated T stretch |
| | 29143 | Deletion | 1 | A | - | 151 | 4594 | 3.287 | 0.381 | 36.728 | Artefact repeated A stretch |
| | 45848 | Deletion | 3 | TAG | - | 153 | 5202 | 2.941 | 0.434 | 37.013 | Artefact related to TAG repeats |
| | 55296 | Deletion | 1 | A | - | 161 | 4978 | 3.234 | 0.495 | 37.366 | Artefact repeated region |
| | 68638 | Deletion | 2 | AC | - | 200 | 3684 | 5.429 | 0.424 | 36.700 | Artefact repeated region |
| | 91564 | Deletion | 3 | GAC | - | 89 | 4095 | 2.173 | 0.418 | 36.921 | Artefact repeated region |
| | 94483 | Deletion | 1 | T | - | 298 | 5559 | 5.361 | 0.422 | 36.990 | Artefact repeated T stretch |
| | 94483 | Insertion | 1 | - | T | 117 | 5491 | 2.131 | 0.452 | 37.179 | Artefact repeated T stretch |
| | 97943 | Insertion | 1 | - | A | 513 | 544 | 94.301 | 0.499 | 36.690 | Artefact repeated region. |
| | 97943 | Insertion | 1 | - | C | 27 | 544 | 4.963 | 0.444 | 37.333 | Artefact repeated region |
| | 97944 | SNV | 1 | T | G | 540 | 544 | 99.265 | 0.494 | 36.157 | Artefact repeated region. |
| | 98204 | SNV | 1 | C | A | 416 | 2995 | 13.890 | 0.436 | 35.007 | Authentic |
| | 113154 | Insertion | 1 | - | T | 1961 | 5016 | 39.095 | 0.436 | 37.303 | Artefact repeated T stretch |
| | 115768 | Deletion | 3 | TAC | - | 166 | 6201 | 2.677 | 0.476 | 37.404 | Artefact related to TAC repeats |
| | 119268 | Deletion | 2 | CA | - | 177 | 5444 | 3.251 | 0.492 | 36.836 | Artefact repeated region |
| | 120114 | Deletion | 1 | T | - | 177 | 6079 | 2.912 | 0.477 | 37.249 | Artefact repeated T stretch |
